# Synthesis and Evaluation of HSOD/PSF and SSOD/PSF Membranes for Removal of Phenol from Industrial Wastewater

**DOI:** 10.3390/polym13081253

**Published:** 2021-04-13

**Authors:** Rivoningo Ngobeni, Olawumi Sadare, Michael O. Daramola

**Affiliations:** 1 Faculty of Engineering and the Built Environment, School of Chemical and Metallurgical Engineering, University of the Witwatersrand, Private Bag X3, Wits, Johannesburg 2050, South Africa; rivoningo.ngobeni.rn@gmail.com; 2 Department of Chemical Engineering, Faculty of Engineering, Built Environment and Information Technology, University of Pretoria, Hatfield, Pretoria 0028, South Africa; wumisadare@gmail.com

**Keywords:** composite membrane, filtration, silica sodalite, hydroxy sodalite, phenol-containing water

## Abstract

Phenol is regarded as a major pollutant, as the toxicity levels are in the range of 9–25 mg/L for aquatic life and humans. This study embedded silica sodalite (SSOD) and hydroxy sodalite (HSOD) nanoparticles into polysulfone (PSF) for enhancement of its physicochemical properties for treatment of phenol-containing wastewater. The pure polysulfone membranes and sodalite-infused membranes were synthesized via phase inversion. To check the surface morphology, surface hydrophilicity, surface functionality, surface roughness and measure the mechanical properties of the membranes, characterization techniques such as Scanning Electron Microscope (SEM), contact angle measurements, Fourier Transform Infrared, Atomic Force Microscopy (AFM) nanotensile tests were used, respectively. The morphology of the composite membranes showed incorporation of the sodalite crystals decreased the membrane porosity. The results obtained showed the highest contact angle of 83.81° for pure PSF as compared to that of the composite membranes. The composite membranes with 10 wt.% HSOD/PSF and 10 wt.% SSOD/PSF showed mechanical enhancement as indicated by a 20.96% and 19.69% increase in ultimate tensile strength, respectively compared to pure PSF. The performance evaluation of the membranes was done using a dead-end filtration cell at varied feed pressure. Synthetic phenol-containing wastewater was prepared by dissolving one gram of phenol crystals in 1 L of deionized water and used in this study. Results showed higher flux for sodalite infused membranes than pure PSF for both pure and phenol-containing water. However, pure PSF showed the highest phenol rejection of 93.55% as compared to 63.65% and 64.75% achieved by 10 wt.% HSOD/PSF and 10 wt.% SSOD/PSF, respectively. The two sodalite infused membranes have shown enhanced mechanical properties and permeability during treatment of phenol in synthetic wastewater.

## 1. Introduction

Phenols and other phenolic compounds are prevalent in various industrial effluents such as resin manufacturing, plastics, paper, paint, wood oil, petroleum refining, coal processing, petrochemicals, pharmaceuticals, and coking operations [1]. United States Environmental Protection Agency (EPA) and the National Pollutant Release Inventory (NPRI) of Canada have designated phenol as a priority pollutant. International regulatory bodies have put stringent discharge limits for phenols [2]. Therefore, discharging phenolic compounds without proper treatment poses a serious health hazard to animals, humans, and aquatic life. EPA has regulated water purity standards for less than 1 ppb for phenol in surface water. The phenol toxicity levels are in the range of 9–25 mg/L for aquatic life and humans [3]. Exposure to phenol for a longer period can lead to irregular breathing, respiratory arrest, and tremor and muscle weakness in humans [3]. Chronic effects due to long-term phenol exposure include weight loss, diarrhea, anorexia, vertigo, salivation, vertigo, salivation, and dark colorations of the urine [3,4]. To proffer a solution to pollution and the health issues posed by the disposal of phenolic compounds in industrial effluents, several processes have been developed.

Traditional methods used to remove phenol like steam distillation, wet air oxidation, adsorption, electrochemical oxidation, liquid–liquid, solid-phase extraction, liquid–liquid extraction, and catalytic wet air oxidation show high phenol removal efficiency. However, they use excessive chemicals [5]. Additionally, advanced technologies used for phenolic treatment such as photo-oxidation, ozonation, UV/H_2_O_2_, and Fenton reaction are costly [6]. Conversely, membrane technology classified among the advanced technologies for phenol treatment is eco-friendly and relatively cheaper [2,6].

Researchers have shifted focus towards the application of membrane for treatment of phenol-containing wastewater due to its cost-effectiveness and absence of chemical additives. However, the main drawback is the flux decline caused by membrane fouling [2]. Membrane fouling is the phenomenon caused by the accumulation of biological species, inorganic, organic and colloidal, onto the membrane surface or within its pores. It results in flux decline and a rapidly increasing transmembrane pressure during operation, and the possible deterioration of mechanical strength [7,8]. To minimize fouling, various methods—alteration of concentration of feed solution, surface modification, and changing the operational conditions—are being developed continuously by researchers [9].

Polymers and ceramics are commonly used in the fabrication of membranes which are used for filtration purposes. Polymer membranes are relatively cheap, but have lower tolerance to harsh conditions, whereas ceramic membranes have higher mechanical strength which results in greater resistance to harsh environments and longer lifespan [10,11]. The wastewater industries have been extensively using polymeric and ceramic membranes for wastewater treatment; hence, the pros and cons of the two membranes are clearly understood [12]. Researchers are exploring effective ways of combining the advantageous features of the two membranes, polymeric and ceramic, to produce a composite material called mixed matrix membrane (MMM) [11]. This membrane is developed to enhance the overall performance of the membrane which would not otherwise be achieved by just using one of the two membranes [13].

MMMs are prepared through dispersion of porous particles, such as silica, carbon molecular sieves, carbon nanotubes (CNTs), and zeolites within a continuous polymer matrix such as polyethersulfone, polysulfone, and polyamide [14,15]. This has proven to enhance mechanical properties, selectivity, and permeability of the polymeric membranes as compared to the equivalent pure polymeric membranes [16]. The synthetic hydroxy sodalite zeolites have proven to have high adsorption capacity. Hence, several researchers propose them as potential fillers in MMM for wastewater treatment [17].

Hydroxy sodalite zeolite, has attractive features for application of selective wastewater treatment such as its high adsorption capacity and very small pores such that only tiny molecules; water (2.65 Å), helium (2.6 Å), and ammonia (2.5 Å), can access and pass through the hydroxy sodalite pores making it a good candidate for wastewater treatment [18]. However, occluded organic matter present in the cages of hydroxy sodalite could result in lower membrane permeation. An attempt to get rid of organic matter through dehydration of the sodalite results in the destabilization and partial collapse of framework that makes sodalite structure [19]. Silica sodalite synthesized via topotactic conversion of a silicate layer as a precursor is formed without organic matter, and therefore has accessible micropores [20]. Silica sodalite as a filler in the polymeric matrix could be the candidate that might out-perform the hydroxy sodalite in terms of membrane flux since it has more accessible pore space for permeation [21]. Therefore, there is a need for a cheap and reliable treatment method that could be used to safely remove hazardous contaminants such as phenols to a safer level.

This study focuses on fabrication of fouling-resistant MMM for treatment of phenol-containing wastewater consisting of polysulfone as the matrix base and silica sodalite as a filler. As comparison, a similar membrane was prepared using hydroxy sodalite (HSOD) nanoparticles obtained via hydrothermal synthesis method. The concentration of the sodalite crystals in the matrix has been kept at 10 wt.% in this study, following the study published by Daramola et al. [22] where 10 wt.% concentration of hydroxy sodalite in PES membrane had shown the highest flux during the AMD treatment. Therefore, 10 wt.% concentration of nanoparticles has desirable flux for this study and has shown good mechanical strength. However, there are limited studies on the application of hydroxy sodalite infused composite membranes and the silica sodalite infused composite membranes for treatment of phenol-containing water. Therefore, this study reports the separation performance of fabricated membranes (hydroxy sodalite infused PSF and silica sodalite infused PSF) during the treatment of phenol-containing wastewater.

## 2. Materials and Methods

Sodium metasilicate nonahydrate (Na_2_SiO_3_), sodium hydroxide pellets (NaOH, 99%), anhydrous sodium aluminate (NaAlO_2_, 99%), Tetra-ethoxysilane, aqueous solution tetramethylammonium hydroxide (25 wt.%: 32 mL), polysulfone (in beaded form with a molecular weight of 22,000 g/mol), *N*,*N*-dimethylacetoamide (˃99%), phenol (99% or more molecular weight 94.11 g/mol) were purchased from Sigma Aldrich (Merck), South Africa and used as supplied without any further purification. The nitrogen gas was purchased from AFROX, South Africa. The de-ionized water was prepared in the laboratory at the School of Chemical and Metallurgical Engineering, University of the Witwatersrand, South Africa.

Hydroxy sodalite crystals were prepared via hydrothermal synthesis method as described by Daramola et al. [22] and were used for comparison purposes. Sodium metasilicate nonahydrate (1.64 g), sodium hydroxide pellets (9.44 g), anhydrous sodium aluminate (0.44 g), and deionized water (44.87 g) were put in a Teflon cup. The Teflon cup was sealed with parafilm sheet, materials were mixed together and stirred for 1 h at 1000 rpm on a magnetic stirrer to yield a homogenous mixture of 5SiO_2_·50NaO_2_·1005HO. During hydrothermal synthesis 45 mL (or 48 g) of the vigorously mixed precursor solution was poured into a Teflon-lined stainless-steel autoclave and subjected to hydrothermal synthesis at 413 K for 3.5 h. After hydrothermal synthesis, hydroxy sodalite crystals were washed thoroughly with deionized water until the pH of the water was neutral. The washed crystals were collected on filter paper and dried overnight at 333 K in an oven.

To synthesize RUB-15, silica sodalite was produced through topotactic conversion using modified version of the method described by Moteki et al. [20]. Tetra-ethoxysilane (18.52 g) was slowly added to an aqueous solution tetramethyl ammonium hydroxide (25 wt.%: 32 mL) in a Teflon cup. The mixture was magnetically stirred for 24 h in order to make it homogenous. The solution was poured into a Teflon-lined stainless-steel autoclave and subjected to the temperature of 413 K for 7 days. The solid particles were filtered and washed with acetone. The solid particles were dried in the oven at 333 K for 24 h.

RUB-15 (0.1 g) was dissolved in 30 mL of 5 M propionic acid. The solution was stirred for 3 h at room temperature. The solid particles were filtered and washed with distilled water and dried in the oven at 333 K for hours. The particles were calcined at 1073 K for 5 h. Silica sodalite nanoparticles in 10 wt.% were added to 50 mL of N, N-dimethylacetamide. The mixture was ultrasonicated for 15 min and agitated for another 15 min. Ten grams of polysulfone was added to the mixture and ultrasonicated for 15 min. Thereafter, stirred on a magnetic stirrer for 24 h at 400 rpm. Phase inversion method was used for the preparation of asymmetric membranes in this study. The solution was cast on a glass plate with the aid of a casting blade. The cast solution was left under ambient conditions for 10 s and thereafter was fully immersed in distilled water for 24 h. The membrane was dried in an oven at 60 °C for 20 min. As for comparison, a similar membrane was prepared with the same procedure, however, using hydroxy sodalite nanoparticles.

To prepare phenol-containing water, 1 g of phenol crystals was dissolved in 1 L of deionized water, the solution was gently shaken to obtain homogenous stock solution. Thereafter, 20 mL of the stock solution was diluted to 1 L with deionized water (20 mL of diluted stock solution equals 20 mg/L).

### 2.1. Characterizations Nanoparticles and Membranes

The crystallinity of the nanoparticles was checked using (X-ray diffractometer) XRD using CoKα radiation (λ = 0.179 nm) at a scan rate of 0.25 s per step and a step size of 0.02°, respectively. The morphology of the synthesized HSOD and SSOD was evaluated with scanning electron microscopy (SEM) (Zeiss model). The nanoparticles were coated with gold-palladium and subjected to the SEM for observation. The surface chemistry of the nanoparticles was checked with FT-IR spectroscopy using Perkin Elmer spectrum two model in the range of 400–4000 wave number cm^−1^. The textural properties of the nanoparticles were evaluated using Brunauer–Emmett–Teller (BET) using Micromeritics Tristar 3000 model surface area and porosity analyzer (Micromeritics Instrument Corp., 4356 Communications Drive, Norcross, GA, USA).

The surface morphology of the synthesized HSOD/PSF and SSOD/PSF membranes as well as cross-section of the fabricated membranes were checked using SEM. The membranes were coated with gold-palladium and subjected to the SEM for observation. The wettability of the membranes was determined through contact angle measurements using dataphysics (OCA 15 model, Filderstadt, Southern Germany). This is the angle a liquid creates with the solid when it is deposited on it [23]. Contact angle was measured by placing water droplets on the outer surface of the membrane using a micro-syringe. The process was repeated 10 times at different positions after which the average value for each sample was reported and recorded for the investigation of the surface hydrophilicity of the membranes [24]. The mechanical properties of the fabricated membranes were analyzed using texture analyzer, (TA.XT.plus model, TA, Stable Micro System, Surrey, UK). Nanotensile tests were done to measure the largest force that fabricated membranes can withstand before breaking apart [24], and characterized the ability of the membranes to resist elastic deformation under tension. The surface roughness measurements were done using (AFM Vi300 model) to evaluate surface properties of the membranes. Roughness values could reveal membrane’s tendency to foul during operation [25].

### 2.2. Performance Evaluation of the Fabricated Membranes

The performance of the membranes was evaluated using dead-end filtration cell. The dead-end filtration set-up consist of a filtration cell where feed was poured, inert nitrogen gas inlet pipe, stirrer bar, and pressure gauge. The set-up was fitted with one membrane at a time during the separation. Deionized water was used as pure water. Pure water permeation through the membrane was used to determine the original flux of the membrane for fouling monitoring [26]. The system was equilibrated by pressurizing pure water on the membrane placed in the cell at 4 bar, this allows the pores to open and gives easy passage of water [27]. The nitrogen gas was used to apply pressure into the filtration cell and pressure varied from 4 to 7 bar. A stirrer plate together with magnetic bar in the filtration cell were used to maintain a homogeneous solution. Permeate from the filtration cell was collected in a measuring cylinder. The analysis of the feed and permeate was done using a pre-calibrated High-Performance Liquid Chromatography (HPLC) model: Agilent 1200 series model, Eclipse XDB C-18 column. Acetonitrile was used as a mobile phase; 600 mL acetonitrile was diluted with 400 mL of deionized water, 10 µL injection volume, and flow rate at 1 mL/min. Pure water flux and phenol-containing flux for the membrane were calculated using Equation (1). The rejection of the membrane was obtained using Equation (2):

(1) $$J=\frac{V}{A},\;$$

where ***J*** is the permeate flux (L/m^2^·h); ***V***, the volumetric flow (L/h); and ***A***, the effective membrane area (m^2^).

(2) $$R=\frac{C_{F}-C_{P}}{C_{F}}\;\times \;100\;\%$$

*R* is the membrane rejection expressed in percentage; *C_F_* is the phenol concentration in the feed stream (mg/L); and *C_P_* is the phenol concentration in the permeate stream from dead-end cell (mg/L).

## 3. Results and Discussion

### 3.1. Crystallinity and Phase of SSOD and HSOD Crystals

**Figure 1:**
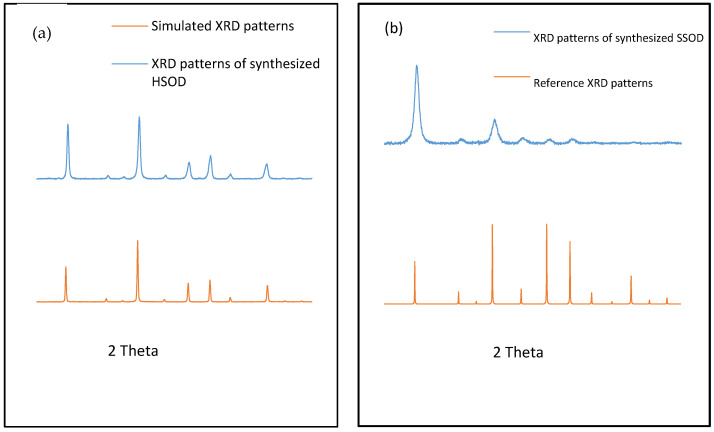
XRD patterns of (**a**) synthesized hydroxy sodalite (HSOD) and simulated XRD patterns, (**b**) synthesized silica sodalite (SSOD) XRD patterns and reference XRD patterns obtained from the International Zeolite Association (IZA) website [28].

Figure 1 depicts XRD patterns of the synthesized HSOD which revealed sharp peaks that are attributable to the crystallinity of the particles. The XRD patterns of the synthesized HSOD match the simulated XRD patterns, this confirms the successful formation of HSOD crystals [22]. The XRD patterns of the synthesized SSOD in Figure 1b reveal the successful formation of SSOD crystals as the patterns match the reference SSOD patterns obtained from the International Zeolite Association (IZA) website [28]. The broad hump at around 2θ = 25, which is a characteristic peak for amorphous silica sodalite, also confirms that the SSOD is successfully formed [29].

### 3.2. Morphology of Sodalite Crystals and Membranes

**Figure 2:**
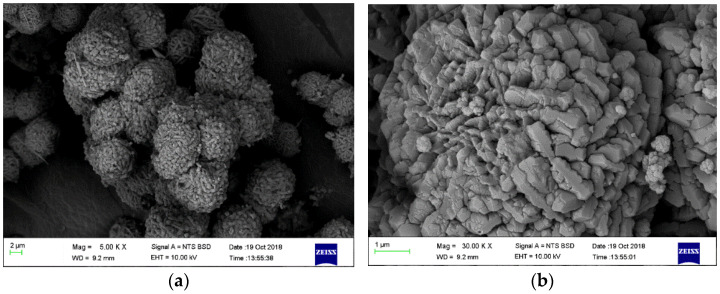
Surface morphology of hydroxy sodalite: (**a**) low magnification (5000× magnification (5.00 kx) and (**b**) high magnification (50,000×).

Figure 2a depicts surface morphology of the synthesized HSOD particles at lower magnification. Thread-ball-like shapes were observed and are consistent with the work reported by Hums [30]. Figure 2b shows SEM image of HSOD at high magnification, typical cubic shapes of the crystals are visible, this further reaffirms the successful formation of HSOD. Similar observations have been reported in the literature [22].

**Figure 3:**
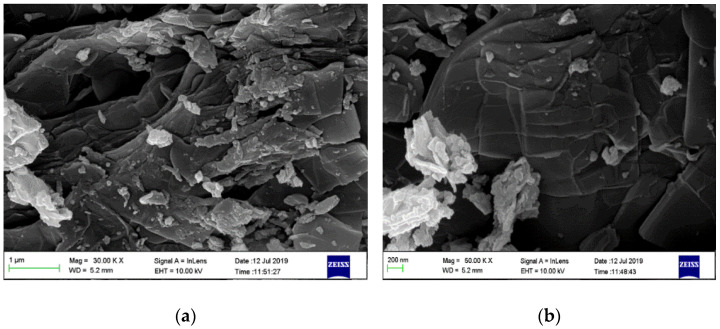
Surface morphology of silica sodalite: (**a**) low magnification (30.00 KX) and (**b**) high magnification (50.00 KX).

Figure 3a shows SEM image of SSOD, thin plate-like shapes stacked together are typical shapes of silica sodalite formed in a topotactic conversion manner. The observation is consistent with the work reported by Koike et al. [29].

Table 1 presents the textural physicochemical properties of the synthesized HSOD SSOD nanoparticles. The results illustrate that, HSOD has a very small specific surface area 2.35 (m^2^/g) compared to the surface area of SSOD 27.29 (m^2^/g), this shows that synthesis via topotactic conversion produces significantly enhanced specific surface area. The larger specific surface area implies increased adsorption capacity, hence more particles from wastewater may be trapped on the surface of the SSOD than HSOD [31,32]. SSOD has larger pore volume than HSOD implying that SSOD has more accessible pores than HSOD. This may consequently give higher permeation flux during performance evaluation as described by Moteki et al. [20].

**Figure 4:**
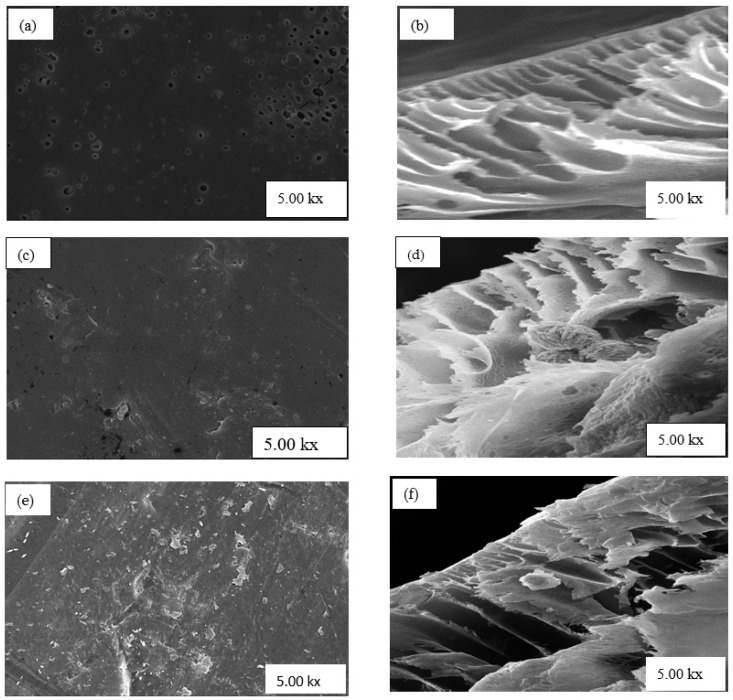
SEM images (**a**,**b**) show surface and cross-section of 0 wt.% NPs/polysulfone (PSF), (**c**,**d**) show surface and cross-section of 10 wt.% HSOD/PSF, and (**e**,**f**) show surface and cross-section of 10 wt.% SSOD/PSF, respectively.

Figure 4a,b depict the surface and cross-section morphology of PSF membrane with no nanoparticles (NPs) (0 wt.% NPs/PSF), respectively. The image in Figure 4a shows large visible pores on the surface of the nanoparticles as well on the cross-section in Figure 4b. Furthermore, the cross-sectional SEM image in Figure 4b shows the asymmetric nature of PSF, whereby the upper layer of the membrane is thin and dense, whereas pores at the bottom are larger. These results also agree with the literature [33]. Figure 4c,d depict the surface view and the cross-sectional view of PSF embedded with 10 wt.% HSOD, respectively. The surface view shows fewer pores which are smaller in comparison with those of the pure PSF membrane, attributable to the suppression of micro-void formation at high percentage loading of nanoparticles within the polymer matrix [34]. The typical thread ball-like shape of the HSOD particles are visible in the cross-section of the PSF membrane. This typical thread ball-like shape of HSOD particles has been reported in the literature [30], thereby confirming the presence of HSOD particles within the polymer. Figure 4e,f depict the surface view and cross-section of the 10 wt.% SSOD/PSF membrane, respectively. The surface view of the membrane shows that the membrane is denser with no visible pores. Attributable to the suppression of micro-void formation caused by high concentration of SSOD particles. The cross-section of the membrane reveals a plate-like shape, indicating the presence of SSOD in the membrane because the shape of SSOD was previously described as plate-like by Koike et al. [29].

### 3.3. Surface Properties of Sodalite Crystals and Membranes via FT-IR

**Figure 5:**
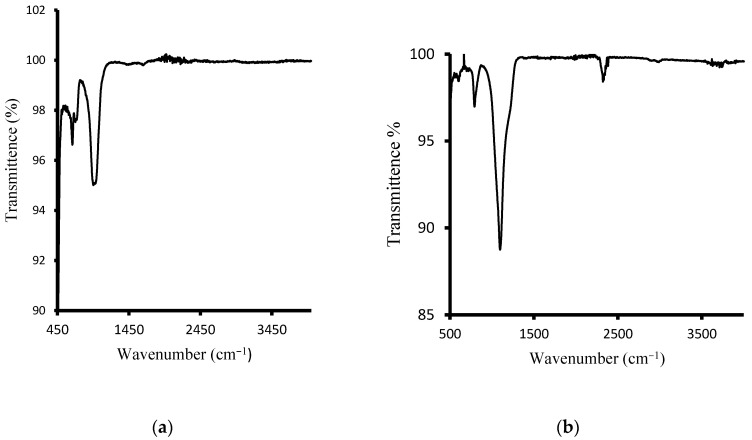
(**a**) Fourier Transform Infrared spectrum of HSOD crystals and (**b**) SSOD nanoparticles.

The surface chemistry of the hydroxy sodalite has been checked with FT-IR spectroscopy. Figure 5a depicts the spectrum of HSOD crystals. The OH-band in the range between 3000 and 4000 cm^−1^ is absent. This has been observed in the literature for basic hydroxy sodalite. However, non-basic hydroxy sodalite with 2 to 8 mol of water is expected to exhibit OH-band [35,36]. The strong broad band peak at approximately 1000 cm^−1^ could be attributed to the asymmetric stretching vibration of T−O−T (T can either be Si or Al). The symmetric stretching vibration at around 740 and 600 cm^−1^ is attributable to T−O−T. The results obtained from the FT-IR analysis of the HSOD show consistency with the literature [22,37]. Figure 5b depicts the spectrum of SSOD nanoparticles. The vibration peak at 1100 cm^−1^ is assignable to Si−O−S asymmetric stretching mode. There is a noticeable absence of the broad absorption band at around 3300 cm^−1^ that belongs to O–H stretching vibration of silanol groups, this is due to the dehydration that takes place during calcination [29].

### 3.4. Surface Roughness of Fabricated Membranes

**Figure 6:**
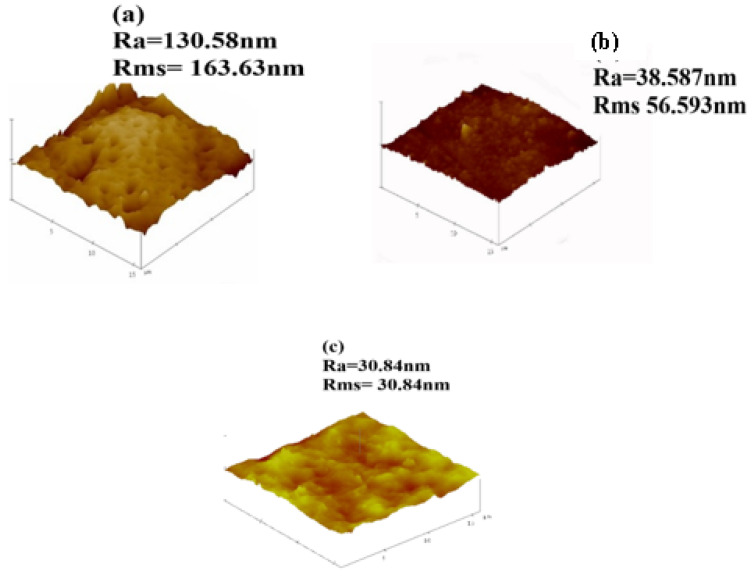
AFM images: (**a**) 0 wt.% NPs/PSF, (**b**) 10 wt.% HSOD/PSF, and (**c**) 10 wt.% SSOD/PSF.

Surface of fibrous composite membrane is rough, this has an influence on the physicochemical properties of fibrous composite membrane. The surface roughness is an important property of fibrous composite membrane and understanding this property is essential to understanding the performance of the membranes [38]. Figure 6 shows 3D images obtained from AFM (together with the values of the surface roughness). Roughness values are used as an indicator of a membrane’s tendency to foul during filtration [25]. When roughness is high, adhesive forces on the membrane surface become larger [39]. The adhesive forces cause particles from wastewater to adhere on the membrane surface making rougher membranes highly susceptible to fouling [40]. The 0 wt.% NPs/PSF membrane has shown the highest average roughness (*R*a) of 163.86 nm and root mean square roughness (*R*ms) 130.58 nm. This could be attributed to the hydrophobic nature of pure polysulfone. The decrease in roughness has been observed when a pure PSF membrane was either loaded with 10 wt.% HSOD or 10 wt.% SSOD nanoparticles. This is due to the changing of roughness characteristics upon addition of hydrophilic nanoparticles that altered the hydrophobic surface of pure PSF to the hydrophilic surface [25]. The 10 wt.% SSOD has shown the least surface roughness 30.84 and 45.84 nm for Ra and Rms, respectively.

### 3.5. Contact Angle Measurements of Membranes

**Figure 7:**
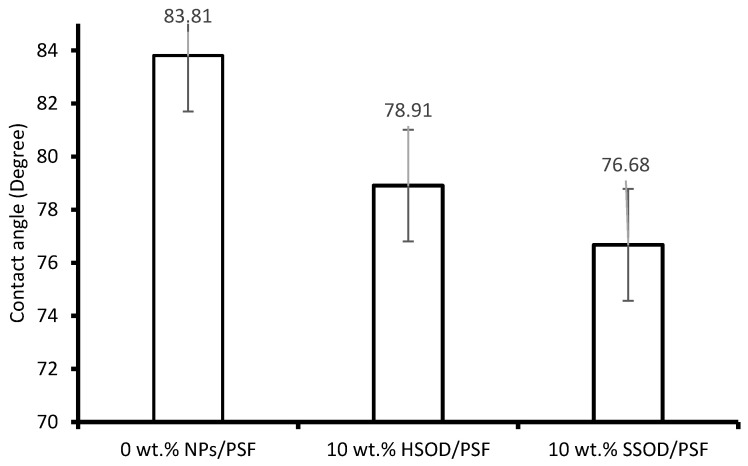
Contact angle of the fabricated membranes.

Figure 7 shows contact angle measurements done to investigate the surface hydrophilicity (wettability by water) of the membranes. This could be used in predicting the performance of the membranes or susceptibility to fouling [24]. The hydrophobic membrane tends to have higher contact angle and hydrophilic membrane have lower contact angle [41]. The pure PSF (0 wt.% NPs/PSF) showed the highest contact angle (83.81°) of all the membranes this is attributed to the hydrophobic nature of the polysulfone membrane, membrane with higher contact angle exhibits poor wettability with water when compared to the one with a lower contact angle [42]. The incorporation of hydroxy sodalite and silica sodalite particles into a pure PSF matrix resulted in a decrease in contact angle. The decrease in the contact angle reveals the enhancement of the hydrophilic property of PSF membrane. This is in agreement with the study reported by Unuigbe et al. [34]. In addition, this observation corroborate the trend observed with the surface roughness of these membranes.

### 3.6. Mechanical Properties of Fabricated Membranes

**Figure 8:**
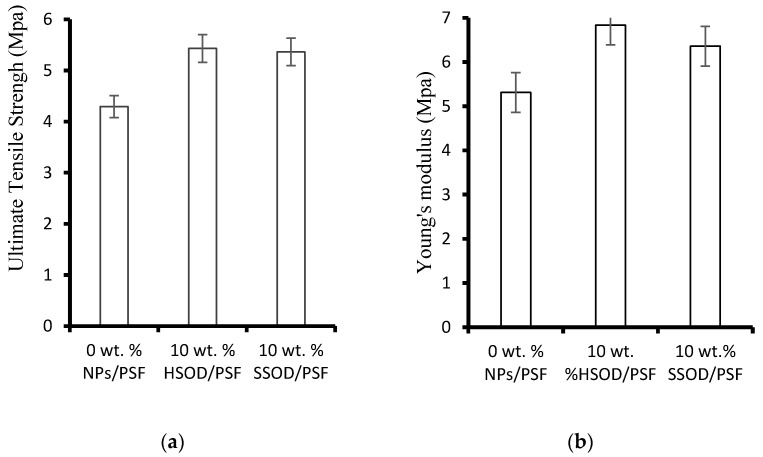
Mechanical strength of the membranes: (**a**) ultimate tensile strength (UTS) and (**b**) Young’s modulus.

Figure 8 depicts the effect of nanoparticles loading on the mechanical properties of the fabricated membranes. Figure 8a shows ultimate tensile strength (UTS) results which show the largest force that fabricated membranes can withstand before breaking apart [24]. Figure 8b shows Young’s modulus which evaluates the ability of the membranes to resist elastic deformation. The research shows that polymer membranes with enhanced mechanical strength may have longer lifespan and less prone to mechanical deterioration [35]. The composite membrane loaded with 10 wt.% HSOD nanoparticles (10 wt.% HSOD/PSF) and the one loaded with 10 wt.% SSOD nanoparticles (10 wt.% SSOD/PSF) displayed enhanced mechanical strength a 20.96% increase in the ultimate tensile strength by 10 wt.% HSOD/PSF and a 19.69% increase in ultimate tensile strength by 10 wt.% SSOD/PSF) when compared to that of the pure PSF. There was a noticeable increase in both the UTS and the Young modulus of the PSF upon the addition of HSOD and SSOD particles, this thereby indicating the enhancement of the mechanical strength of PSF [11,22]. Enhancement of the mechanical strength of PSF is instrumental to elongating the life span and integrity of these membranes while it prevents them from mechanical deterioration compared to the pure PSF [35].

### 3.7. Performance Evaluation of the Membranes during Treatment of Phenol-Containing Wastewater

**Figure 9:**
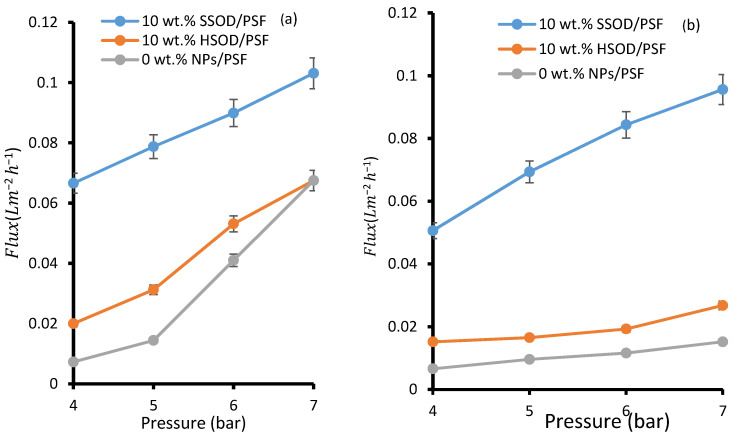
Membrane flux: (**a**) pure water flux and (**b**) phenol-containing water.

Figure 9a depicts the pure water flux—deionized water was used as pure water. The pure water flux for 0 wt.% NPs/PSF membrane was lower than that obtained from 10 wt.% HSOD/PSF and from 10 wt.% SSOD/PSF membranes. This observation could be attributed to the hydrophilic nature of the NPs embedded within the PSF membrane. These particles attract water molecules towards them resulting in higher permeation flux [43]. When pressure was increased to 7 bar, there was an increase in flux for 0 wt.% NPs/PSF membrane which was even higher than the one obtained for the 10 wt.% HSOD/PSF membrane at the same pressure. This observation could be attributed to the opening of free fractional volume of the polymer matrix at a higher pressure as PSF is highly porous, but water repels at lower pressure due to its hydrophobic surface [44]. The 10 wt.% SSOD/PSF membrane displayed the highest pure water flux. This may be due to the accessibility of pores of SSOD nanoparticles embedded in PSF and its hydrophilicity which attracts water molecules toward it. Figure 9b shows flux during the treatment of phenol-containing wastewater, flux is relatively lower as compared to pure water for all the membranes. This indicates fouling and competitive sorption between phenol and water molecules [27]. In the case of pure water flux membrane, resistance depends only on the pore structures of the membrane [45,46]. Increasing the pressure increases the driving force for permeation flux, resulting in the higher permeation flux for both pure water and phenol-containing wastewater [27]. The increase in flux upon addition of nanoparticles in this study is consistent with the reported literature by Maphutha et al. [47]. However, Maphutha et al. [47] reported flux as high as ~325 Lm^−2^h^−1^, but a relatively low flux is recorded in this study. Lower flux recorded in this study could be attributed to higher concentration of PSF at 20% (*w*/*v*) as compared to 10% (*w*/*v*) from the study conducted by Maphutha et al. [47], as higher concentration reduces membrane porosity.

**Figure 10:**
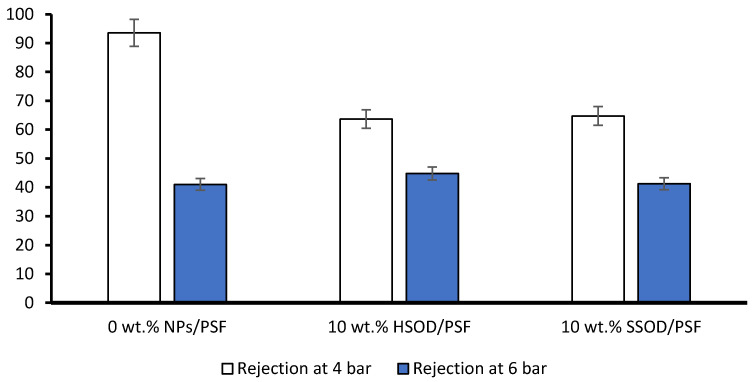
Phenol rejection from 0 wt.% NPs/PSF, 10 wt.% HSOD/PSF, and 10 wt.% HSOD.

Figure 10 depicts phenol rejection from pure polysulfone (0 wt.% NPs/PSF) and composite membranes 10 wt.% HSOD/PSF and 10 wt.% SSOD/PSF. Phenol molecules are only expected to permeate through membrane pores when the pressure on the filtration cell exerts a force much greater than the opposing capillary force on the surface of the membrane [48]. The pure polysulfone showed the highest rejection of 93.55% at 4 bar. This may be due to slightly thicker membrane or polysulfone may be synthesized with slightly more concentration than the composite membranes [45]. However, at higher pressure (6 bar) there was a huge decrease in phenol rejection to 41%, this resulted in 56.17% rejection loss, which may be caused by opening of membrane pores. The 10 wt.% HSOD/PSF and 10 wt.% SSOD/PSF composite membranes showed 63.65% and 64.75% phenol rejection, respectively at 4 bar. However, at 6 bar, the rejection of 10 wt.% HSOD/PSF and 10 wt.% SSOD/PSF decreased to 44.8% and 41.25%, respectively. The composite membranes depict relatively lower rejection loss of 29.62% for 10 wt.% HSOD/PSF and 36.29% for 0 wt.% SSOD/PSF as compared to 56.17% for pure polysulfone. The composite membranes are more sustainable at high pressure. This could be attributed to the incorporation of nanoparticles in the membrane matrix, which reduced the porosity of the membrane [49]. Hence, the composite membranes do not severely lose their rejection capacity.

Table 2 presents the comparison of results in this study with the literature. The two sodalite membranes synthesized for this study performed relatively well in comparison with sodalite infused membranes in the literature. Daramola et al. [22] investigated the application of hydroxy sodalite in polyethersulfone (PES-SOD) mixed matrix membrane for AMD treatment. They fabricated PES-SOD mixed matrix with different loadings together with pure polymeric membrane for comparison. The results showed that the performance of a pure polymeric membrane (selectivity and flux) is enhanced by loading hydroxy sodalite crystals within the matrix of PES polymer. The results of membrane performance evaluation showed that the 10 wt.% has higher flux, whereas 15 wt.% loading showed highest selectivity towards Pb^2+^ (57.44% rejection).

## 4. Conclusions

Novel mixed matrix membranes 10 wt.% SSOD/PSF and 10 wt.% HSOD/PSF have been developed successfully via phase inversion method. The 10 wt.% HSOD/PSF membrane was synthesized and used for comparison purposes in this present study. The two sodalite infused membranes have shown enhanced mechanical properties and permeability during treatment of phenol from synthetic wastewater. There was no significant difference between HSOD and SSOD infused membranes in terms of rejection. However, SSOD infused membrane showed highest permeation flux. The permeability and selectivity for SSOD/PSF membrane is slightly higher than HSOD/PSF membrane. From this study it cannot be safely concluded that SSOD has better performance than HSOD. Therefore, a novel mixed matrix membrane with enhanced mechanical properties and selectivity was successfully developed for removal of phenol from industrial wastewater.

## Figures and Tables

**Table 1:** BET analysis of HSOD synthesized via the hydrothermal method and SSOD synthesized via topotactic conversion.

Nanoparticles	BET Surface Area (m^2^/g)	Pore Volume (cm^3^/g)	Pore Size (nm)
**HSOD**	2.35	0.0119	20.29
**SSOD**	27.29	0.0672	9.84

**Table 2:** The comparison of the performance between HSOD/PSF and SSOD/PSF and literature.

Membrane	Permeate Flux (L/m^2^h)	Wastewater Contaminants	% Rejection	References
1 wt.% fCNT/PSF/PVA	-	Phenol	79. 21	[27]
10 wt.% CNT/PSF/PVA	-	Oily Wastewater	97.39	[47]
4 wt.% CNT/PSF	~12	Phenol	42.98	[49]
10 wt.% HSOD/PSF	0.0152	Phenol	63.65	This study
10 wt.% SSOD/PSF	0.0506	Phenol	64.75	This study

## Data Availability

Not applicable.
